# Evaluation of an AIDS Educational Mobile Game (AIDS Fighter · Health Defense) for Young Students to Improve AIDS-Related Knowledge, Stigma, and Attitude Linked to High-Risk Behaviors in China: Randomized Controlled Trial

**DOI:** 10.2196/32400

**Published:** 2022-01-24

**Authors:** Jian Tang, Yu Zheng, Daiying Zhang, Xingli Yu, Jianlan Ren, Mei Li, Yue Luo, Min Tian, Yanhua Chen

**Affiliations:** 1 Department of Operating Room The Affiliated Hospital of Southwest Medical University Luzhou China; 2 Department of Rheumatology and Immunology The Affiliated Hospital of Southwest Medical University Luzhou China; 3 Department of Anesthesiology The Affiliated Hospital of Southwest Medical University Luzhou China; 4 School of Nursing Southwest Medical University Luzhou China; 5 Department of Nursing The Affiliated Hospital of Southwest Medical University Luzhou China

**Keywords:** young students, AIDS education, educational game, game-based intervention, serious games, public health, HIV, AIDS epidemic, stigma, health defense, health knowledge, digital health, digital health intervention

## Abstract

**Background:**

The AIDS epidemic among young students is serious, and effective preventive interventions are urgently needed. Game-based intervention has become an innovative way to change healthy behaviors, and we have developed an AIDS educational game called AIDS Fighter · Health Defense.

**Objective:**

In this study, we tested the effect of AIDS Fighter · Health Defense on young students in improving AIDS-related knowledge, stigma, and attitude related to high-risk behaviors in Southwest China.

**Methods:**

A randomized controlled trial was conducted from September 14 to 27, 2020. In total, 96 students from 2 classes in a middle school were selected by stratified cluster sampling in Luzhou City, Southwest China. The students were randomly divided into the intervention group (n=50, 52%) and the control group (n=46, 48%). The intervention group played the AIDS educational game AIDS Fighter · Health Defense; the control group learned AIDS-related knowledge through independent learning on the QQ chat group. An AIDS-related knowledge questionnaire, a stigma scale, and an attitude questionnaire on AIDS-related high-risk behaviors were used to measure the effect of the AIDS educational game via face-to-face interviews. The user experience of the game was assessed using the Educational Game User Experience Evaluation Scale. The difference was statistically significant at *P*≤.05.

**Results:**

After the intervention, the AIDS knowledge awareness rate (X̅ [SD], %) of the intervention and control groups were 70.09 (SD 11.58) and 57.49 (SD 16.58), with *t*=4.282 and *P*<.001. The stigma scores of the 2 groups were 2.44 (SD 0.57) and 2.48 (SD 0.47), with *t*=0.373 and *P*=0.71. The positive rate (X̅ [SD], %) of attitudes of high-risk AIDS behaviors of the 2 groups were 82.00 (SD 23.44) and 79.62 (SD 17.94), with *t*=0.555 and *P*=0.58. The mean percentage of the game evaluation was 54.73% as excellent, 31.45% as good, 13.09% as medium, and 0.73% as poor.

**Conclusions:**

AIDS Fighter · Health Defense could increase AIDS-related knowledge among young students, but the effect of the game in reducing AIDS-related stigma and improving the attitudes of high-risk AIDS behaviors was not seen. Long-term effects and large-scale studies are needed to assess the efficacy of game-based intervention.

**Trial Registration:**

Chinese Clinical Trial Registry ChiCTR2000038230; https://trialsearch.who.int/Trial2.aspx?TrialID=ChiCTR2000038230

## Introduction

### Importance of AIDS Prevention Education for Young Students

According to the Joint United Nations Programme on HIV/AIDS (UNAIDS) data, there were approximately 3.4 million HIV infections among young people aged 15-24 years worldwide and approximately 460,000 new HIV infections among young people in 2019 [1], most of them being students, and we are far away from reaching UNAIDS testing and condom use targets of 95% coverage by 2030 [2].

Therefore, it is necessary to take effective measures to control the prevalence of AIDS among young students [3]. Studies have shown that AIDS health education for young students can improve their ability to prevent AIDS [4-6], but traditional education methods are not attractive to them, and the effect is poor [7,8]. Therefore, it is urgent to innovate the AIDS education model to improve the education effect and curb the spread of AIDS among young students.

### Importance of AIDS Educational Games for AIDS Prevention

With the development of science and technology, AIDS prevention education has changed from using traditional methods to using modern ones [9,10]. Among them, game-based intervention has become an innovative way to change healthy behaviors [11-13]. Educational games contain mechanisms such as tasks, rules, feedback, challenges, and components such as points, badges, and leaderboards [14], which may influence people’s motivation and behavior by stimulating their immersion, satisfaction, and experience [15].

Research on AIDS health education games started late, mainly aimed at adolescents, including HIV-infected adolescents, gay men and other high-risk groups, and healthy adolescents. The targets of education are to increase adolescents' adherence to antiviral therapy and pre-exposure prophylaxis (PrEP) [16-18], promote HIV screening [19], reduce adolescent risky sex and multiple sexual partners, avoid drug and alcohol abuse, and thus reduce the infection of AIDS and sexually transmitted diseases among adolescents [20-22]. The main types of games are role-playing, online interaction, knowledge contest, hero combat, etc, and the main game mechanisms are challenge, task, upgrade, interaction, virtual, etc. [23-26].

### AIDS Educational Games in China

AIDS health educational games in China are in their infancy, and with few existing studies, single game elements, incomplete game mechanisms, and a lack of effect evaluation studies, the actual educational effect is not clear. To solve these problems, we developed an AIDS educational game, mainly targeting education for HIV/AIDS knowledge, attitude, and behavior, and refusal of drug and alcohol abuse. The game has a variety of elements and mechanisms that are suitable for AIDS prevention education of adolescents in China.

In this study, we tested the effectiveness of the AIDS educational game on preventing HIV among young students in Luzhou City, Southwest China. Here we report results from this study.

## Methods

### Game Design

The AIDS educational game is called AIDS Fighter · Health Defense. The story line is that HIV launches an attack on the human body and players need to control heroes to eliminate HIV. During the battle, players are repeatedly trained to take condoms and refuse dangerous sexual behaviors, avoid drugs to refuse intravenous drug use, avoid alcohol to refuse dangerous sex when drunk, obtain antiviral drugs for PrEP and postexposure prophylaxis (PEP) [27].

The human body system is used as the level in the game. There are 7 levels in total. The difficulty of the game increases from low to high. The sequence of levels is (1) immune system, (2) blood system, (3) skin and mucosal system, (4) central nervous system, (5) respiratory system, (6) digestive system, and (7) genitourinary system.

The game copywriting of each level corresponds to the human body system of the level, describing that the system is infected with HIV. The success or failure of the game has corresponding text prompts. If the game fails, it prompts HIV to invade the human body and shows what the result will be; if it succeeds, encouraging text appears to remind the player that they have successfully avoided HIV infection.

AIDS Fighter · Health Defense has the following mechanisms and components: (1) a virus combat game, (2) goal setting to eliminate HIV, (3) questions to be answered to be resurrect in the game, (4) point ranking, and (5) recognition and rewards. The game includes 5 functional modules:

- Game module: There are 7 levels involving 7 systems of the human body affected by AIDS.
- Quiz-and-Answer module: It provides a chance to the player to resurrect by answering questions on AIDS-related knowledge.
- Knowledge Corner module: It contains educational articles and videos on AIDS.
- Point-ranking module: Points are awarded according to the behavior and clearance situation during the game and the learning in the Knowledge Corner module, and the points are ranked.
- Data Management module: The educational information in the Knowledge Corner module can be edited, and in-game data collection, integration, classification, and application can be realized.

**Figure 1:**
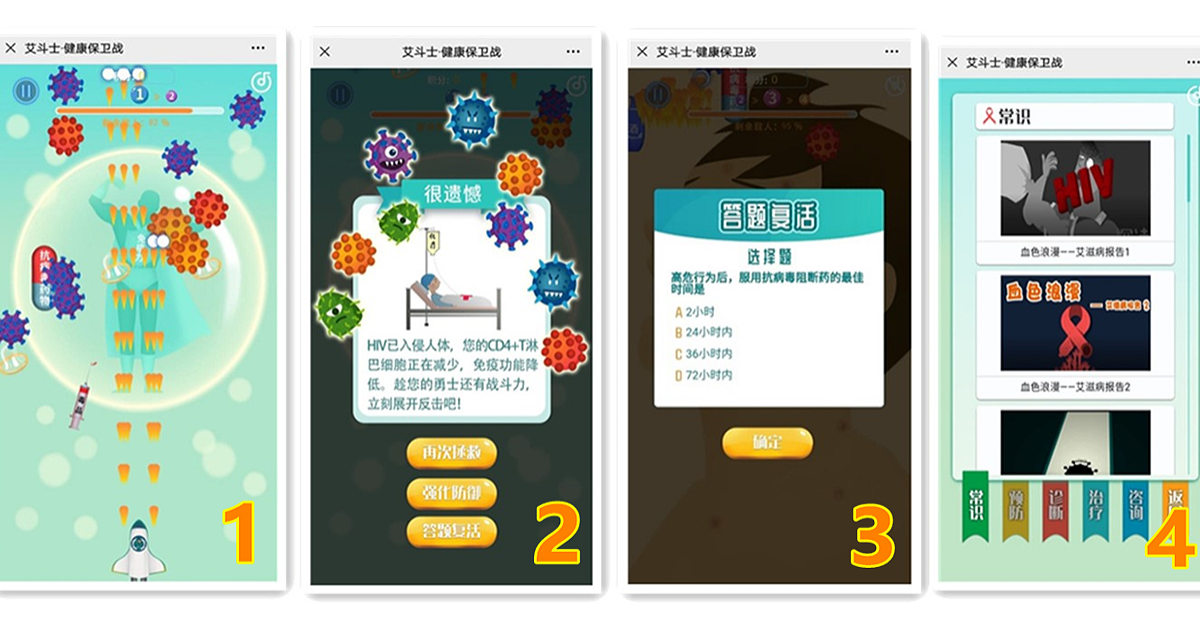
Gameplay and screenshots of AIDS Fighter · Health Defense. Interface 1 is the gameplay. Interface 2 is the screenshot of game failure. Interface 3 is the Quiz-and-Answer module. Interface 4 is the Knowledge Corner module.

The gameplay and screenshots are shown in Figure 1.

### Study Design

A randomized controlled trial was conducted, in which 2 classes were selected from a middle school in Luzhou City using stratified cluster sampling. Third-party staff randomly allocated the 2 classes to the intervention group and the control group by tossing a coin.

We had the following hypotheses (H):

- H1: AIDS Fighter · Health Defense can increase the awareness of AIDS-related knowledge among young students.
- H2: AIDS Fighter · Health Defense can reduce AIDS-related stigma among young students.
- H3: AIDS Fighter · Health Defense can improve the attitude of AIDS-related high-risk behaviors among young students.

The study was approved by the ethics committee of the Affiliated Hospital of Southwest Medical University, China.

### Participants

The participants of the study were first-grade students of a secondary vocational school, and the location was Luzhou City, Southwest China. The eligibility criteria for participation were as follows: young students aged 15-24 years, informed consent and voluntary participation in this study (<16 years old required the consent of the guardian), and ability to access the internet. The exclusion criteria were as follows: The participant or guardian refused to provide consent to participate in the study, the participant did not complete the questionnaire survey, and the participant had used similar games before.

### Intervention

The intervention and control groups received the corresponding intervention from September 14 to 27, 2020.

The intervention group played AIDS Fighter · Health Defense through WeChat (social software that can also provide some games). Participants were asked to play the game and study the AIDS-related knowledge in the Knowledge Corner module and were required to earn at least 20 points per day. The details of the participants' use of the game can be checked through the game’s data management system.

Participants in the control group were added to the QQ chat group (QQ software is one of the most popular social software programs in China, which supports online chat, video chat, and voice chat; file sharing; network hard disk; remote control; email; online teaching; and other functions; Shenzhen Tencent Computer Systems Company Limited). The same educational resources as the Knowledge Corner module in AIDS Fighter · Health Defense were uploaded to the QQ group. Participants were asked to learn the knowledge by themselves.

### Outcomes and Measurements

The primary outcomes were AIDS-related knowledge, stigma, and attitude before and 14 days after intervention.

AIDS-related knowledge was assessed through a self-made questionnaire with a total of 45 items, including the following 6 dimensions: basic knowledge of AIDS, knowledge of AIDS prevention, knowledge of AIDS testing, knowledge of AIDS treatment, knowledge of AIDS PrEP, and knowledge of AIDS-related laws and regulations.

AIDS-related stigma was assessed through the Chinese version of the Zelaya HIV/AIDS Stigma Scale [28] with 24 items, including 6 positive items, 18 negative items. The positive items have a score of 1 for totally agree to 5 for totally disagree, and the negative items have a score of 1 for totally disagree to 5 for totally agree. The items are divided into the following 4 dimensions: fear of transmission and disease; association with shame, blame, and judgment; personal support of discriminatory actions or policies; and perceived community support of discriminatory actions or policies. Each dimension contains 6 items with a score of 1 for totally agree to 5 for totally disagree. The higher the score, the more the participant’s stigma of AIDS.

AIDS-related attitude was assessed through a questionnaire with 8 items with the choice of “Yes” or “No” in Chinese. Five experts were invited to verify the content validity of these questionnaires, and the item-level content validity index (I-CVI) and scale-level CVI (S-CVI) were both 1.00.

The secondary outcome was the user's experience of the game in the intervention group. It was assessed through the Educational Game User Experience Evaluation Scale with 22 items in Chinese. After playing the game, the user selects one of 4 options (excellent, good, medium, and poor) to evaluate each indicator according to their actual experience.

### Data Analysis

The classification data were statistically described by percentage, the qualitative data were compared using the chi-square test, the statistical description of the quantitative data was given using by *X̅* (SD), the two-tailed *t* test was performed to compare the groups, and difference-in-difference analysis was used to accurately evaluate the change in the intervention group. The difference was statistically significant when *P*≤.05. The data analysts were blinded to the allocation.

## Results

### Description of Study Sample

The 96 students included were aged from 16 to 19 years. There were no significant demographic differences between the 2 groups (Table 1).

**Table 1:** Participant demographics (N=96).

Characteristics			Intervention group (n=50), n (%)		Control group (n=46), n (%)		*t/χ*^2^ (*df*=94)		*P* value
Age (years), mean (SD)			16.68 (0.74)		16.76 (0.71)		0.547		0.59
**Gender**									
	Male	21 (42)		21(45.65)		0.130		0.72	
	Female	29 (58)		25(54.35)		0.130		0.72	
**Ethnicity**									
	Han	49 (98)		43(93.48)		0.356		0.55	
	Other ethnic groups	1 (2)		3 (6.52)		0.356		0.55	
**Place of residence**									
	Rural area	29 (58)		32 (69.57)		1.383		0.24	
	Urban areas	21 (42)		14 (30.43)		1.383		0.24	
**Growth environment**									
	Living with parents	22 (44)		29 (63.04)		6.873		0.15	
	Living with mother	7 (14)		8 (17.39)		6.873		0.15	
	Living with father	6 (12)		1 (2.17)		6.873		0.15	
	Living with grandparents	11 (22)		5 (10.87)		6.873		0.15	
	Other	4 (8)		3 (6.52)		6.873		0.15	
**Access to AIDS information^a^**									
	TV	37 (74)		38 (82.61)		1.712		0.19	
	Newspaper	11 (22)		12 (26.09)		0.250		0.62	
	Internet	44 (88)		39 (84.78)		0.373		0.54	
	WeChat	19 (38)		17 (36.96)		0.014		0.91	
	WeiBo	18 (36)		16 (28.26)		0.019		0.89	
	School education	38 (76)		31 (67.39)		1.369		0.24	
	Medical staff	25 (50)		18 (39.13)		1.473		0.23	
	Relatives, friends	20 (40)		11 (23.91)		3.376		0.07	
	Other	1 (2)		3 (6.52)		0.896		0.34	

^a^Multiple choices available.

### Comparison of AIDS-Related Knowledge, Stigma, and Attitude Between the 2 Groups Before the Intervention

Before the intervention, there was no statistically significant difference in the AIDS knowledge awareness rate, stigma scores, and positive rates of attitude of high-risk IDS behaviors between the 2 groups (Table 2).

**Table 2:** Comparison of indicators between the 2 groups before intervention (N=96).

Items		Intervention group (n=50), mean (SD)		Control group (n=46), mean (SD)		*t* (*df*=94)		*P* value
**AIDS knowledge awareness rate**								
	Overall	58.04 (17.55)	57.87 (11.80)		0.056		0.96	
	Basic knowledge of AIDS	64.77 (17.10)	65.55 (15.19)		0.236		0.81	
	Knowledge of AIDS prevention	55.20 (25.97)	51.74 (16.37)		0.787		0.43	
	Knowledge of AIDS testing	48.80 (24.96)	47.83 (22.50)		0.200		0.84	
	Knowledge of AIDS treatment	68.00 (31.15)	65.76 (27.06)		0.377		0.71	
	Knowledge of AIDS PrEP^a^	35.20 (18.76)	32.17 (19.54)		0.774		0.44	
	Knowledge of AIDS-related laws and regulations	65.75 (27.87)	71.47 (21.03)		1.141		0.26	
**Stigma score**								
	Overall	2.58 (0.61)	2.51 (0.49)		0.622		0.54	
	Fear of transmission and disease	2.93 (1.13)	2.97 (0.96)		0.187		0.85	
	Association with shame, blame, and judgment	2.47 (0.82)	2.31 (0.70)		1.031		0.31	
	Personal support of discriminatory actions or policies	2.72 (0.74)	2.58 (0.68)		0.966		0.34	
	Perceived community support of discriminatory actions or policies	2.20 (0.82)	2.16 (0.65)		0.266		0.79	
**Positive rate of attitude of high-risk AIDS behaviors**								
	Overall	77.50 (22.73)	85.05 (19.30)		1.747		0.08	
	Do you support premarital sex?	72.00 (45.40)	80.00 (40.10)		0.917		0.36	
	Do you support premature love?	52.00 (50.50)	57.00 (50.10)		0.487		0.63	
	Do you support a 1-night stand?	88.00 (32.80)	96.00 (20.60)		1.443		0.15	
	Will you have premarital sex with your boyfriend/girlfriend?	74.00 (44.30)	80.00 (40.10)		0.697		0.49	
	Will you have sex with someone other than your lover?	82.00 (38.80)	87.00 (34.10)		0.650		0.52	
	Will you have sex with the same gender?	88.00 (32.80)	89.00 (31.50)		0.152		0.88	
	Will you use condoms when you have sex with your lover?	80.00 (40.40)	93.00 (25.00)		1.912		0.06	
	Will you use condoms when you have sex with other people of the same/opposite sex?	74.00 (44.30)	80.00 (40.00)		0.697		0.49	

^a^PrEP: pre-exposure prophylaxis.

### Comparison of AIDS-Related Knowledge, Stigma, and Attitude Between the 2 Groups After the Intervention

After the intervention, the awareness rate of AIDS-related knowledge in the intervention group was higher than that in the control group (*t*=4.282, *P*<.001). The stigma scores of the 2 groups were not statistically significant (*t*=0.373, *P*=.71). The positive rates of attitude of high-risk AIDS behaviors in the 2 groups were not statistically significant (*t*=0.555, *P*=.58); see Table 3.

**Table 3:** Comparison of indicators between the 2 groups after intervention (N=96).

Items			Intervention group (n=50), mean (SD)		Control group (n=46), mean (SD)		*t* (*df*=94)		*P* value
**AIDS** **knowledge awareness rate**									
	Overall	70.09 (11.58)		57.49 (16.58)		4.282		<0.001	
	Basic knowledge of AIDS	74.62 (13.84)		64.38 (17.04)		3.243		0.002	
	Knowledge of AIDS prevention	66.20 (18.83)		52.61 (22.15)		3.247		0.002	
	Knowledge of AIDS testing	62.00 (25.64)		42.61 (25.86)		3.685		<0.001	
	Knowledge of AIDS treatment	83.00 (21.09)		72.28 (30.84)		2.002		0.05	
	Knowledge of AIDS PrEP^a^	44.40 (17.75)		26.52 (20.25)		4.609		<0.001	
	Knowledge of AIDS-related laws and regulations	82.25 (15.79)		73.64 (20.79)		2.296		0.02	
**Stigma score**									
	Overall	2.44 (0.57)		2.48 (0.47)		0.373		0.71	
	Fear of transmission and disease	2.59 (0.86)		2.85 (0.95)		1.407		0.16	
	Association with shame, blame, and judgment	2.55 (0.83)		2.34 (0.51)		1.478		0.14	
	Personal support of discriminatory actions or policies	2.45 (0.67)		2.54 (0.61)		0.686		0.49	
	Perceived community support of discriminatory actions or policies	2.15 (0.73)		2.17 (0.63)		0.143		0.89	
**Positive rate of attitude of high-risk AIDS behaviors**									
	Overall	82.00 (23.44)		79.62 (17.94)		0.555		0.58	
	Do you support premarital sex?	84.00 (37.00)		76.00 (43.10)		0.978		0.33	
	Do you support premature love?	64.00 (48.50)		59.00 (49.80)		0.498		0.62	
	Do you support a 1-night stand?	92.00 (27.40)		98.00 (14.70)		1.320		0.19	
	Will you have premarital sex with your boyfriend/girlfriend?	82.00 (38.80)		74.00 (44.40)		0.942		0.35	
	Will you have sex with someone other than your lover?	86.00 (35.10)		89.00 (31.50)		0.439		0.66	
	Will you have sex with the same gender?	94.00 (24.00)		98.00 (14.70)		0.974		0.33	
	Will you use condoms when you have sex with your lover?	84.00 (37.00)		70.00 (46.50)		1.639		0.11	
	Will you use condoms when you have sex with other people of the same/opposite sex?	80.00 (40.00)		74.00 (44.40)		0.697		0.49	

^a^PrEP: pre-exposure prophylaxis.

### Comparison of AIDS-Related Knowledge, Stigma, and Attitude Before and After Intervention in the 2 Groups

After the intervention, the AIDS knowledge awareness rate of the intervention group was higher than that before the intervention (*t*=4.052, *P*<.001). The difference in the AIDS stigma score was not statistically significant (*t*=1.186, *P*=.24) after the intervention. The difference in the positive rate of attitude of high-risk AIDS behaviors was also not statistically significant (*t*=0.975, *P*=.33) after the intervention. After the intervention in the control group, the AIDS knowledge awareness rate, the stigma score, and the positive rate of attitude of high-risk AIDS behaviors were not statistically significant, but the positive rate of “Will you use condoms when you have sex with your lover?” dropped from 93.00 (SD 25.00) to 70.00 (SD 46.50), with *t*=2.955 and *P*=.004; see Table 4.

**Table 4:** Comparison of indicators before and after intervention in the 2 groups (N=96).

Items			Intervention group (n=50)							Control group (n=46)					
		Before intervention, mean (SD)		After intervention, mean (SD)	*t* (*df*=49)		*P* value		Before intervention, mean (SD)		After intervention, mean (SD)	*t* (*df*=45)		*P* value	
**AIDS knowledge awareness rate**															
	Overall	58.04 (17.55)		70.09 (11.58)	4.052		<0.001		57.87 (11.80)		57.49 (16.58)	0.127		0.90	
	Basic knowledge of AIDS	64.77 (17.10)		74.62 (13.84)	3.166		0.002		65.55 (15.19)		64.38 (17.04)	0.348		0.73	
	Knowledge of AIDS prevention	55.20 (25.97)		66.20 (18.83)	2.425		0.017		51.74 (16.37)		52.61 (22.15)	0.214		0.83	
	Knowledge of AIDS testing	48.80 (24.96)		62.00 (25.64)	2.608		0.011		47.83 (22.50)		42.61 (25.86)	1.033		0.30	
	Knowledge of AIDS treatment	68.00 (31.15)		83.00 (21.09)	2.820		0.006		65.76 (27.06)		72.28 (30.84)	1.078		0.28	
	Knowledge of AIDS PrEP^a^	35.20 (18.76)		44.40 (17.75)	2.519		0.013		32.17 (19.54)		26.52 (20.25)	1.362		0.18	
	Knowledge of AIDS-related laws and regulations	65.75 (27.87)		82.25 (15.79)	3.642		<0.001		71.47 (21.03)		73.64 (20.79)	0.498		0.6280	
**Stigma score**															
	Overall	2.58 (0.61)		2.44 (0.57)	1.186		0.239		2.51 (0.49)		2.48 (0.47)	0.300		0.77	
	Fear of transmission and disease	2.93 (1.13)		2.59 (0.86)	1.693		0.094		2.97 (0.96)		2.85 (0.95)	0.603		0.55	
	Association with shame, blame, and judgment	2.47 (0.82)		2.55 (0.83)	0.485		0.629		2.31 (0.70)		2.34 (0.51)	0.235		0.82	
	Personal support of discriminatory actions or policies	2.72 (0.74)		2.45 (0.67)	1.913		0.059		2.58 (0.68)		2.54 (0.61)	0.297		0.77	
	Perceived community support of discriminatory actions or policies	2.20 (0.82)		2.15 (0.73)	0.22		0.748		2.16 (0.65)		2.17 (0.63)	0.075		0.94	
**Positive attitude rate of high-risk AIDS behaviors**															
	Overall	77.50 (22.73)		82.00 (23.44)	0.975		0.332		85.05 (19.30)		79.62 (17.94)	1.398		0.17	
	Do you support premarital sex?	72.00 (45.40)		84.00 (37.00)	1.449		0.151		80.00 (40.10)		76.00 (43.10)	0.461		0.65	
	Do you support premature love?	52.00 (50.50)		64.00 (48.50)	1.212		0.228		57.00 (50.10)		59.00 (49.80)	0.192		0.85	
	Do you support a 1-night stand?	88.00 (32.80)		92.00 (27.40)	0.662		0.510		96.00 (20.60)		98.00 (14.70)	0.536		0.59	
	Will you have premarital sex with your boyfriend/girlfriend?	74.00 (44.30)		82.00 (38.80)	0.961		0.339		80.00 (40.10)		74.00 (44.40)	0.680		0.49	
	Will you have sex with someone other than your lover?	82.00 (38.80)		86.00 (35.10)	0.541		0.590		87.00 (34.10)		89.00 (31.50)	0.292		0.77	
	Will you have sex with the same gender?	88.00 (32.80)		94.00 (24.00)	1.044		0.299		89.00 (31.50)		98.00 (14.70)	1.756		0.08	
	Will you use condoms when you have sex with your lover?	80.00 (40.40)		84.00 (37.00)	0.516		0.607		93.00 (25.00)		70.00 (46.50)	2.955		0.004	
	Will you use condoms when you have sex with other people of the same/opposite sex?	74.00 (44.30)		80.00 (40.00)	0.711		0.479		80.00 (40.00)		74.00 (44.40)	0.681		0.49	

^a^PrEP: pre-exposure prophylaxis.

### Mean Change in AIDS-Related Knowledge, Stigma, and Attitude Before and After Intervention in the 2 groups

The mean differences between the intervention and control groups before the intervention (baseline differences) were not statistically significant. After the intervention, the AIDS knowledge awareness rate of the intervention group was higher than that of the control group, and the difference-in-difference of the two groups was significant (*P*=.004), but the difference in the knowledge of AIDS testing and AIDS treatment was not significant. The mean differences in stigma scores and positive rates of attitude of high-risk AIDS behaviors were not statistically significant (Table 5).

**Table 5:** Mean change in indicators before and after intervention in the 2 groups (N=96).

Items		Intervention group (n=50)			Control group (n=46)							
		Before intervention, mean (SD)	After intervention, mean (SD)	Before intervention, mean (SD)		After intervention, mean (SD)	Baseline difference^a^ *P* value		Difference-in-difference,^b^ mean (SD)		*P* value	
**AIDS knowledge awareness rate**												
	Overall	58.04 (17.55)	70.09 (11.58)	57.87 (11.80)		57.49 (16.58)	0.96		12.40 (4.23)		0.004	
	Basic knowledge of AIDS	64.77 (17.10)	74.62 (13.84)	65.55 (15.19)		64.38 (17.04)	0.81		11.02 (4.58)		0.02	
	Knowledge of AIDS prevention	55.20 (25.97)	66.20 (18.83)	51.74 (16.37)		52.61 (22.15)	0.43		10.13 (6.13)		0.100	
	Knowledge of AIDS testing	48.80 (24.96)	62.00 (25.64)	47.83 (22.50)		42.61 (25.86)	0.85		18.42 (7.16)		0.01	
	Knowledge of AIDS treatment	68.00 (31.15)	83.00 (21.09)	65.76 (27.06)		72.28 (30.84)	0.69		8.48 (8.03)		0.29	
	Knowledge of AIDS PrEP^c^	35.20 (18.76)	44.40 (17.75)	32.17 (19.54)		26.52 (20.25)	0.44		17.88 (3.89)		<0.001	
	Knowledge of AIDS-related laws and regulations	65.75 (27.87)	82.25 (15.79)	71.47 (21.03)		73.64 (20.79)	0.20		14.33 (6.31)		0.02	
**Stigma score**												
	Overall	2.58 (0.61)	2.44 (0.57)	2.51 (0.49)		2.48 (0.47)	0.50		–0.11 (0.16)		0.48	
	Fear of transmission and disease	2.93 (1.13)	2.59 (0.86)	2.97 (0.96)		2.85 (0.95)	0.84		–0.34 (0.29)		0.23	
	Association with shame, blame, and judgment	2.47 (0.82)	2.55 (0.83)	2.31 (0.70)		2.34 (0.51)	0.29		0.05 (0.21)		0.81	
	Personal support of discriminatory actions or policies	2.72 (0.74)	2.45 (0.67)	2.58 (0.68)		2.54 (0.61)	0.33		–0.23 (0.20)		0.25	
	Perceived community support of discriminatory actions or policies	2.20 (0.82)	2.15 (0.73)	2.16 (0.65)		2.17 (0.63)	0.78		–0.05 (0.21)		0.79	
**Positive rate of attitude of high-risk AIDS behaviors**												
	Overall	77.50 (22.73)	82.00 (23.44)	85.05 (19.30)		79.62 (17.94)	0.08		9.93 (6.09)		0.10	
	Do you support premarital sex?	72.00 (45.40)	84.00 (37.00)	80.00 (40.10)		76.00 (43.10)	0.32		16.33 (11.99)		0.18	
	Do you support premature love?	52.00 (50.50)	64.00 (48.50)	57.00 (50.10)		59.00 (49.80)	0.66		9.83 (14.36)		0.49	
	Do you support a 1-night stand?	88.00 (32.80)	92.00 (27.40)	96.00 (20.60)		98.00 (14.70)	0.14		1.83 (7.25)		0.80	
	Will you have premarital sex with your boyfriend/girlfriend?	74.00 (44.30)	82.00 (38.80)	80.00 (40.10)		74.00 (44.40)	0.45		14.52 (12.13)		0.23	
	Will you have sex with someone other than your lover?	82.00 (38.80)	86.00 (35.10)	87.00 (34.10)		89.00 (31.50)	0.49		1.83 (10.13)		0.86	
	Will you have sex with the same gender?	88.00 (32.80)	94.00 (24.00)	89.00 (31.50)		98.00 (14.70)	0.38		–14.70 (7.75)		0.06	
	Will you use condoms when you have sex with your lover?	80.00 (40.40)	84.00 (37.00)	93.00 (25.00)		70.00 (46.50)	0.23		19.91 (11.00)		0.07	
	Will you use condoms when you have sex with other people of the same/opposite sex?	74.00 (44.30)	80.00 (40.00)	80.00 (40.00)		74.00 (44.40)	0.46		12.52 (12.24)		0.31	

^a^*P* value for the mean difference between intervention and control groups before the intervention (baseline differences).

^b^Difference-in-difference. It shows whether the expected mean change in indicators from before to after the intervention was different between control and intervention groups.

^c^PrEP: pre-exposure prophylaxis.

### Game Experience Evaluation of the Intervention Group

The user's evaluation of the game was divided into 4 options: excellent, good, medium, and poor. The mean percentage of the game evaluation was 54.73% as excellent, 31.45% as good, 13.09% as medium, and 0.73% as poor (Table 6).

**Table 6:** Game evaluation details.

Second-level index		Excellent, %	Good, %	Medium, %	Poor, %	
**Perceived beauty first-level index, mean (SD)**						
	Clear interface design	34 (68)	12 (24)	4 (8)	0	
	Reasonable interface menu	29 (58)	17 (34)	4 (8)	0	
	New and interesting interface	28 (56)	13 (26)	8 (16)	1 (2)	
	Beautifully crafted interface	26 (52)	16 (32)	7 (14)	1 (2)	
	Pleasant interface	22 (44)	18 (36)	9 (18)	1 (2)	
**Availability** **first-level index, mean (SD)**						
	Easy to remember	28 (56)	11 (22)	11 (22)	0	
	Easy to play	29 (58)	14 (28)	7 (14)	0	
	Easy to learn	35 (70)	10 (20)	5 (10)	0	
	The game runs stably without faults	24 (48)	10 (20)	13 (26)	3 (6)	
	System feedback is obviously timely and appropriate	28 (56)	16 (32)	6 (12)	0	
**Educational** **first-level index, mean (SD)**						
	Knowledge feedback is clear and timely	32 (64)	15 (30)	3 (6)	0	
	Levels conform to the regular pattern of learning	28 (56)	16 (32)	6 (12)	0	
	Reliable content, flexible and diverse forms	34 (68)	12 (24)	4 (8)	0	
	Clear learning goals	30 (60)	14 (28)	5 (10)	1 (2)	
	Balance of skill and challenge	29 (58)	16 (32)	5 (10)	0	
**Gameplay** **first-level index, mean (SD)**						
	Suitable challenge	21 (42)	17 (34)	12 (22)	0	
	Reasonable incentives	25 (50)	17 (34)	8 (16)	0	
	Optional	20 (40)	25 (50)	5 (10)	0	
	Attractive plot	17 (34)	20 (40)	12 (24)	1 (2)	
	Clear rules of the game	29 (58)	19 (38)	2 (4)	0	
**Learning needs**						
	The learning content of the game meets or exceeds the needs of learners	31 (62)	15 (30)	4 (8)	0	
	The function of the game meets or exceeds the needs of learners	23 (46)	23 (46)	4 (8)	0	

## Discussion

### Effect on AIDS-Related Knowledge of AIDS Fighter · Health Defense Game

After the intervention with the AIDS educational game, the awareness rate of AIDS knowledge of the intervention group was higher than that of the control group, and the differences were statistically significant, which implies AIDS Fighter · Health Defense can improve AIDS-related knowledge of young students and has the potential to improve their AIDS prevention capabilities. However, the difference-in-difference analysis of the knowledge of AIDS testing and treatment was not statistically significant, which was different from the results of the *t* test. The possible reason is that the AIDS-related knowledge received during the intervention period led to the same trend of change in the 2 groups, while the effect of the game intervention was not significant on the knowledge of AIDS testing and treatment. This may also be due to the short intervention time and small sample size, leading to an insignificant intervention effect. Future studies should carry out continuous, large-sample intervention investigations to improve the knowledge of HIV testing and treatment of young students, especially the knowledge of HIV treatment, which is not only important for young students to take PrEP and PEP but also important for people with HIV, especially in low- and middle-income countries, to improve their antiviral treatment adherence [29].

After the intervention group played the AIDS Fighter · Health Defense game, the awareness rate of AIDS knowledge in all dimensions was higher than that before the intervention. Of these dimensions, the awareness rate of AIDS-related laws and regulations increased the most (*t*=3.642, *P*<.001). Due to the low awareness of AIDS-related laws and regulations in the public [30], patients with HIV infection are still being unfairly treated in terms of equal employment, privacy protection, and social discrimination. They not only need to endure the discomfort caused by HIV but also need to face social disapproval and exclusion [31]. We found that AIDS Fighter · Health Defense has a good effect on improving the knowledge of AIDS-related laws and regulations in young students. Further studies could try to apply the game to other groups for AIDS health education and provide educational tools for improving the public’s knowledge of AIDS-related laws and regulations.

There was no significant difference in the awareness rate of AIDS knowledge in the control group after the intervention, which implies that the effect of self-learning via the internet to improve AIDS-related knowledge of young students is poor. Under the background of exam-oriented education represented by China, young students have a strong learning ability to master the key points of learning quickly and effectively before the exam. Therefore, if an examination or assessment to test the learning effect could be added to the self-learning of AIDS health education for young students, it may increase the self-learning effect for them and improve their AIDS-related knowledge.

### Effect on AIDS-Related Stigma of AIDS Fighter · Health Defense

This study found that after the intervention with AIDS Fighter · Health Defense, the AIDS-related stigma of young students showed a downward trend, although the results were not statistically significant. This may be because the formation and change of an individual’s cognition of or attitude toward something is a complex process that takes a long time [32], while the intervention time of this study was 2 weeks, so the results obtained may only have found a trend of decreasing AIDS-related stigma. If long-term intervention can be carried out, the AIDS-related stigma reduction may be more obvious. In addition, the sample size of this study was small, which may also have caused the difference to be not significant enough. AIDS Fighter · Health Defense has shown some effects in reducing AIDS-related stigma among young students. Therefore, it may have the potential to become an effective educational tool to reduce AIDS-related stigma in the public.

### Effect of AIDS Fighter · Health Defense on Attitude of High-Risk AIDS Behaviors

Reducing high-risk AIDS behaviors is one of the effective ways to prevent AIDS [33], and one of our goals of AIDS education is to improve the attitudes of drug injection–related risk behaviors and sexual risk behaviors. Unexpectedly, after 2 weeks of self-learning of AIDS knowledge in the control group, the positive rate of attitudes of high-risk AIDS behaviors decreased, and the most obvious option for the decline in the positive rate was “Will you use condoms when you have sex with your lover?”, which dropped from 93.00 (SD 25.00) to 70.00 (SD 46.50), with *t*=2.955 and *P*=.004. This result suggests that acquiring AIDS-related knowledge through online self-learning is not only less effective in education but may also make young students more open to sexual attitudes and more likely to indulge in risky sexual behaviors. We found that after the intervention with AIDS Fighter · Health Defense, the positive rate of attitudes of high-risk AIDS behaviors of young students increased. Therefore, game-based AIDS education has the potential to become an intervention method to improve the attitudes of high-risk AIDS behaviors and AIDS Fighter · Health Defense could become an effective and large-scale intervention tool.

### Game Experience Evaluation of the Intervention Group

The results of game experience suggest that young students have positive feedback for AIDS Fighter · Health Defense, believing that the game probably has high aesthetics and usability and is educational and playful, which can achieve or exceed the needs of learners. However, there are problems, such as insufficient stability of the game, insufficient novelty of the game elements, and insufficient appeal of the game plot. This reminds us that we should strengthen the combination of education and entertainment in AIDS game education and increase the appeal of educational games on the premise of ensuring the effect of AIDS education.

### Conclusion

This study found that AIDS Fighter · Health Defense can improve the AIDS-related knowledge among young students, but the effect of the game in reducing AIDS-related stigma and improving the attitudes of high-risk AIDS behaviors was not observed. This might be due to the small sample size and short intervention time; the results of AIDS-related stigma and attitudes of high-risk behaviors were not statistically significant. Therefore, large-scale, continuous, and multicenter research is needed to assess the efficacy of game-based intervention.

## Appendix

Multimedia Appendix 1 CONSORT-EHEALTH checklist (V. 1.6.1).

## Abbreviations

CVI: content validity index
I-CVI: item-level content validity index
PEP: postexposure prophylaxis
PrEP: pre-exposure prophylaxis
S-CVI: scale-level content validity index
UNAIDS: Joint United Nations Programme on HIV/AIDS
